# Nitrogen Use Efficiency and Carbon Traits of High-Yielding European Hybrid vs. Line Winter Wheat Cultivars: Potentials and Limitations

**DOI:** 10.3389/fpls.2018.01988

**Published:** 2019-01-17

**Authors:** Lukas Prey, Sebastian Kipp, Yuncai Hu, Urs Schmidhalter

**Affiliations:** Department of Plant Sciences, Technical University of Munich, Munich, Germany

**Keywords:** commercial heterosis, nitrogen allocation and partitioning, nitrogen translocation, phenotyping, yield formation

## Abstract

In contrast to allogamous crops, hybrid wheat has only recently been fostered by breeding companies in Europe. Hybrid cultivars are often associated with higher stress resistance, e.g. under drought conditions, but little is known about the nitrogen (N) use efficiency of modern hybrid wheat cultivars. Therefore, four high-yielding European hybrid and nine line winter wheat (*Triticum aestivum* L.) cultivars were grown under three N regimes in a high-yielding German environment and compared over 3 years at anthesis and maturity for 53 direct and indirect traits of yield formation and N allocation. Dry matter and N uptake were determined on the plant and plant organ levels. Commercial heterosis, expressing the performance of hybrid in comparison to line cultivars, was positive for about one-third of the 53 direct and indirect N and carbon traits. On average, hybrid cultivars yielded more grain (+5.5%), mainly due to a higher harvest index (+3.5%) together with higher post-anthesis assimilation and more grains per spike. However, grain N content was lower for hybrids (−8.5%), so their grain N uptake was not higher. This went along with comparable trait values for N translocation and the temporal N uptake of the different plant organs. Current wheat hybrids seem to be more efficient in overall N use because they are better at converting (higher N utilization efficiency) comparable amounts of N uptake (N uptake efficiency) into grain biomass. The results suggest that given increased seed costs for hybrids, the yield advantage of hybrid cultivars over locally adapted line cultivars will have to be further increased for establishing hybrids in low-stress, high-yielding environments.

## Introduction

In spite of ongoing breeding progress (Mackay et al., 2011; Laidig et al., 2014), yield gains in winter wheat (*Triticum aestivum* L.) have slowed down during the last years (Fischer et al., 2014). This effect was associated with a shift in the acreage percentage (Laidig et al., 2014), input extensification in some Western countries (Lassaletta et al., 2014), but increasingly also with climatic changes (Lobell et al., 2011). This development goes along with higher yield fluctuations (Peltonen-Sainio et al., 2010). At the same time, farmers are facing more and more constraints in the allowed fertilization rates, especially for nitrogen and phosphorus due to stricter environmental legislation (van Grinsven et al., 2012). Nonetheless, the trend of a higher demand for cereals is unbroken (Fischer et al., 2014).

Due to the environmental impacts of excessive nitrogen use, the low overall conversion efficiency of nitrogen into biomass, expressed as nitrogen use efficiency (NUE; Galloway and Cowling, 2002), and the high costs of nitrogen, breeding efforts for fostering NUE need to be increased (Cormier et al., 2013, 2016; Lammerts van Bueren and Struik, 2017). Although plant breeding doubled the nitrogen use efficiency during the last century (Calderini, 1995), further improvements are required.

Hybrid wheat breeding is considered as a potential method to alleviate yield depressions and fluctuations caused by weather extremes and associated plant stress (Mühleisen, 2015). In allogamous crops like rye and corn, substantial yield improvements were achieved from exploiting heterosis effects (Birchler et al., 2010). However, the realized heterosis is substantially lower in autogamous crops like wheat (Morgan et al., 1989; Mahajan et al., 1999; Longin et al., 2012). Moreover, avoiding self-pollination for hybridization is still a major challenge together with achieving sufficient spread of viable pollen (Singh et al., 2010; Longin et al., 2012; Roy and Sarkar, 2013). For various reasons, in spite of decade-old attempts (Singh et al., 2010), hybrid wheat is still not cultivated widely with about a 1% share of the global wheat production (Longin et al., 2012). Nonetheless, with increasing plant breeders' efforts (Fischer et al., 2014) and new molecular and genetic technologies that may become available (Whitford et al., 2013; Zhao et al., 2014; Boeven et al., 2016), the performance of hybrids in comparison to line cultivars as expressed by the “commercial heterosis” may increase in the future.

Given the high costs for hybrid wheat seed production, a sufficiently increased crop output needs to be achieved in order to establish the use of hybrid seeds on the market. Therefore, a reduction in seeding density by up to more than half was suggested for hybrid cultivars in order to reduce seeding costs, depending on the hybrid seed price and the achievable yield advantage of hybrid over line cultivars (Jordaan, 1996; Bodson et al., 1997). However, not all studies found different tolerances for the low seeding densities of hybrid compared to top line cultivars (Lloveras et al., 2004).

Depending on the test conditions and the plant material, the estimated heterosis differed substantially. Thus, higher heterosis was found for the N-fertilized treatment compared to the control treatment (Kindred and Gooding, 2005). Best-parent heterosis for grain yield evaluated in 430 hybrids in England was only 3 to 6% (Morgan et al., 1989). Mid-parent heterosis for the grain yield of experimental hybrids ranged from non-significant effects in one year to up to 12.2% in the other year and was higher for the non-fertilized compared to the fertilized treatment (Le Gouis et al., 2002). Heterosis was found to be higher in drought-stressed environments compared to non-stressed environments (Noorka et al., 2013). In low-yielding drought-influenced environments, Jordaan (1996) found average yield advantages of commercial hybrid over line cultivars in 2 years of 11.5 and 14.8%, respectively, and an increasing relative hybrid advantage with less favorable growing conditions. Based on a substantial yield advantage of up to 26%, hybrid wheat was found to be economically beneficial under Indian conditions (Matuschke et al., 2007).

Yield heterosis could often not be ascribed to a consistent shift in a single yield component (Bodson et al., 1997; Singh et al., 2010) whereas the number of spikelets per spike was earlier stated to best explain yield heterosis (Mahajan et al., 1999).

Currently, increased breeding efforts, especially in dry Chinese regions (Fischer et al., 2014), India (Roy and Sarkar, 2013) and Western Europe, notably in France and Germany, raise the question of the agronomic performance of modern hybrid cultivars.

Longin et al. (2013) assessed a large population of 1,604 hybrids and found mid-parent heterosis of 10.7% for grain yield and commercial yield heterosis for 69 of the hybrids. Following Weissmann and Weissmann (2002), the authors stated that the necessary commercial heterosis of about 1 t ha^−1^ was exceeded by 11 of the hybrids. While heterosis for heading time was very weak, plant height heterosis was also supposed to indicate increased early vigor of hybrids.

A review of 15 earlier studies until the late 1990s gave median yield heterosis values of about 12% compared to reference cultivars (Mahajan et al., 1999). Yield trend analysis of hybrid and line cultivars in the Great Plains over 28 years evidenced higher genetic gain of hybrid cultivars, however, without higher yield stability (Koemel et al., 2004). In a similar environment, the hybrid yield advantage was estimated to be 10.8% and increased under better cropping conditions but without higher stability (Bruns and Peterson, 1998). In contrast, hybrids of wheat, barley and triticale were found to have a more stable yield under West-European conditions (Mühleisen et al., 2014). However, this study was mainly based on experimental, non-commercial hybrids in comparison to their parental lines.

Hybrid cultivars are often associated with an increased stress tolerance, which is occasionally attributed to sturdier root growth (Sinha and Khanna, 1975; Yao et al., 2005; Wang et al., 2006; Song et al., 2007). Assuming improved yield potential and better root growth, hybrid wheat was hypothesized to also be more resource-efficient in terms of nutrient uptake and its conversion into biomass (Kindred and Gooding, 2004). Even if the environmental effect on heterosis is not clear, some evidence was found for heterotic yield improvement under constant grain nitrogen content or vice versa (Cormier et al., 2016). A positive trend from 10 to 15% yield gain over check cultivars was observed for an early generation of hybrid cultivars (Perenzin et al., 1998). Besides yield heterosis, these authors also found an increased protein content in hybrids. Parental heterosis effects were reported both for grain yield and for grain N yield experimental hybrids under French conditions (Oury et al., 1995). While N uptake until anthesis and its translocation into the grain was similar for hybrid and line genotypes, hybrids were found to have higher post-anthesis N uptake (+18%), enhanced (+17%) dry matter accumulation, and partly prolonged grain filling but similar dry matter translocation, remaining vegetative N and grain N content compared to lines. Since no significant increase in grain number per m^2^ was found, yield gain was due to higher kernel weight. In a previous study, heterosis for grain yield strongly depended on the test environment but was generally positive, whereas protein content was in the range of the parents' performance (Oury et al., 1994). Mostly, the negative relationship between these two traits was found to be stronger for hybrid genotypes but still, positive heterosis was found for protein yield, indicating higher grain N uptake efficiency. For flour quality traits such as kernel hardness, mostly no differences between hybrids and lines were identified in this study. Comparing the effects of seeding rate and N fertilization on the components of N use efficiency of three hybrid cultivars and their parents in England, mid-parent heterosis for total N uptake (Nup) was weak, but stronger for grain Nup, indicating an increased N harvest index of hybrids (Kindred and Gooding, 2004). Similarly, no difference for the overall N utilization efficiency (NutEff) was found but for grain NutEff, so that the increased overall N use efficiency was attributed to higher NutEff for grain. The grain N content was partly lower in hybrids than in lines. Contrary to some previous studies, heterosis was lower in the unfertilized treatment so that heterosis was explained by greater growth potential rather than a better resource efficiency per se.

On average, 20 hybrids under Belgian conditions were found to have superior yield performance compared to high-performance commercial line cultivars by around 7%, but there was no consistent advantage in Nup (Bodson et al., 1997). The economic optimum N doses were found to be 20 kg/ha^−1^ lower for one hybrid compared to one line cultivar by these authors.

A recent study assessed the potential of a large set of experimental modern wheat hybrids compared to their parents and check cultivars for grain yield, grain protein content, grain N deviation, and baking quality (Thorwarth et al., 2018). When analyzed by quality classes, hybrids showed often higher grain yield than line genotypes and check cultivars but lower protein content, whereas sedimentation volume as an indicator of baking quality was at similar levels. Still, on average higher protein yield was found, indicating an advantage of hybrids for N uptake efficiency.

Many of the studies we are aware of indicated the advantage of hybrid wheat in comparison to hybrid parent lines, leading to the conclusion that yield and N use efficiency can show substantial heterosis effects. However, line breeding advanced as well and evidence on the commercial heterosis in comparison to commercial cultivars is still scarce, especially under West-European conditions. Therefore, the objective of the present study was to assess the performance of commercial hybrid vs. line winter wheat cultivars with respect to grain yield and nitrogen use efficiency dependent on different nitrogen regimes. Further traits for understanding organ-specific dry matter (DM) and N allocation and their dynamics during the grain filling phase were considered as well.

## Materials and Methods

### Study Site and Experimental Conditions

The field trials were conducted in a split-plot design with N-level on the main plot and genotype on the subplot during three growing seasons in 2013/2014, 2014/2015, and 2015/2016 at the Dürnast research station in southeast Germany (48.406 N, 11.692 E). The fields comprised mostly of homogeneous Cambisol of silty clay loam with a pH of 6.4, K_2_O-content of 12 mg 100 g^−1^, P_2_O_5_-content of 12 mg 100 g^−1^ and C_org_-content of 1.18%. The average annual precipitation in this region is ~800 mm and the average annual temperature 7.8°C. Previous crops were silage corn in the first year and winter wheat in the second and third year, respectively. Therefore, soil nitrogen delivery was low as assessed from an incremental N fertilization experiment conducted directly next to the trials in all years. In the non-N-fertilized treatments, the grain yield amounted to 24, 15, and 37 dt ha^−1^ and the grain N yield to 27, 20, and 44 kg ha^−1^ in 2014, 2015, and 2016, respectively.

**Figure 1:**
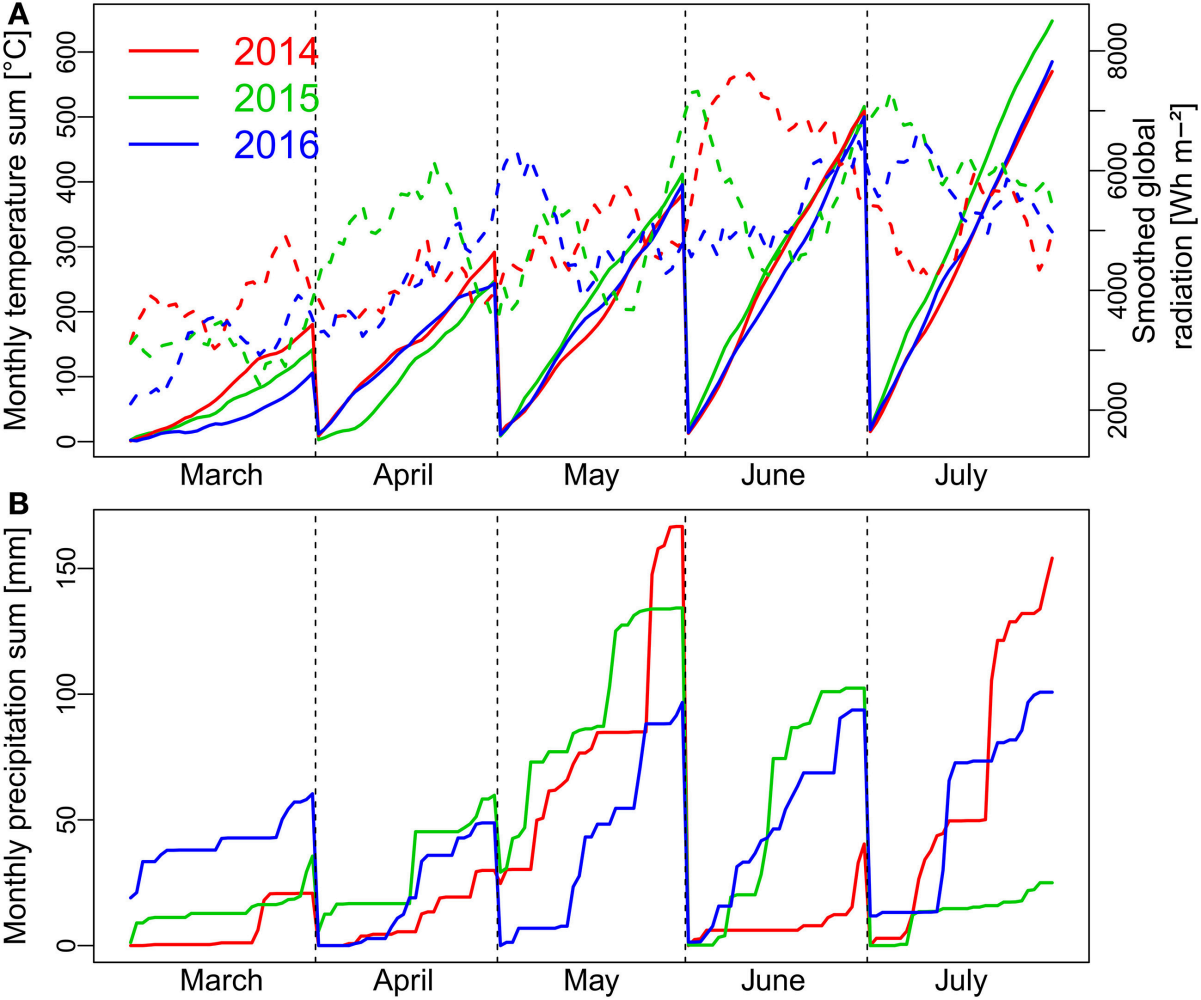
Weather conditions with monthly temperature sum (**A**; continuous lines), daily global radiation (**A**; dashed lines) and monthly precipitation sum **(B)** during the main months of the three growing seasons from March to July. Global radiation is displayed as smoothed by a 10-day moving average. Flowering was from beginning to mid-June in all years.

The year 2014 was characterized by favorable growing conditions at the start of the vegetation in March with a higher temperature sum and more radiation than in the following years, whereas early growth was hampered by cold conditions in 2016 (Figure 1). In spite of the highest global radiation in 2015, April was colder this year but similar both in temperature and radiation in 2014 and 2016. More radiation during early May together with favorable temperatures contributed to accelerated vegetative growth in 2016. In all years, precipitation was sufficient during the vegetative phase until the end of May. However, strong precipitation in May together with unfavorable, wet soil conditions during sowing and pre-winter development led to visible stagnant moisture effects on some plots in 2015. The grain filling phase in 2014 benefited from a high radiation budget in June with still sufficient soil water supply from the precipitation events in May. In contrast, global radiation in June was lowest in 2016 together with lower temperatures. Due to low precipitation and high temperatures, heat and drought stress became apparent during the later grain filling phase in July in 2015.

A set of 13 winter wheat (*Triticum aestivum* L.) cultivars (Table 1) was mechanically drilled to a depth of 3 cm with a row spacing of 12.5 cm at 350 kernels m^−2^. The winter wheat cultivars comprised 7 high-performance commercial German line cultivars and 4 hybrid cultivars and were registered and released by national authorities. Additionally, the two hybrid parental lines “Piko” and “SUR.99820” were included and were in the range of the other line cultivars for grain yield. The cultivars represent all major German quality groups for winter wheat (E, A, B, C) and are mostly frequently grown by German farmers. Sowing dates were October 22, November 4 and October 13 for the first, second and third season, respectively. Plot width was 1.5 m and plot lengths were 11.75, 11.25, and 9.25 m in the first, second, and third year, respectively.

**Table 1:** List of cultivars.

**Cultivar**	**2014**	**2015**	**2016**	**Year of release**	**Cultivar group**	**Quality group**
Anapolis		dh	dh	2013	Line	C
Hybery	dh	dh	dh	2010	Hybrid	B
Hybred	dh	dh	dh	2003	Hybrid	B
Hyland	dh	dh	dh	2009	Hybrid	B
Hystar	dh	dh	dh	2007	Hybrid	B
Impression	dh	dh	dh	2005	Line	A
JB Asano	dh	dh	dh	2008	Line	A
Kerubino	dh	dh	dh	2004	Line	E
Kometus	dh	dh	dh	2011	Line	A
Mulan	dh	dh	dh	2006	Line	B
Patras		dh	dh	2012	Line	A
Piko		dh	dh	1994	Hybrid father line	
SUR.99820	dh	dh	dh		Hybrid mother line	

“*dh” denotes destructively harvested cultivars. The quality groups refer to best baking quality (E), high baking quality (A), sufficient baking quality (B) and feed wheat (C)*.

Each cultivar was grown at three nitrogen fertilization levels (N-level: N1, N2, N3) in 4 replicates per N-level.

Nitrogen as ammonium nitrate granule was applied at tillering (30/60/90 kg N ha^−1^), at stem elongation (30/60/90 kg N ha^−1^) and at heading (40/40/40 kg N ha^−1^) in N1, N2, and N3, respectively. Due to shifted phenology, N fertilization took place on different dates: February 27, March 19 and March 22 for the tillering dressing, April 15, May 11 and April 29 for the dressing during stem elongation and May 20, June 11, and May 23 for the final dressing in 2014, 2015, and 2016, respectively.

An adequate supply of K, P, and S was ensured, and integrated pest management kept the trials weed-free. Depending on pathogen pressure, foliar fungicide was applied 4 times in 2014, 3 times in 2015 and 2 times in 2016. According to local practice, Chlormequat-containing straw-shortener was applied in all years to avoid lodging, and insecticides were used against cereal leaf beetles.

### Plant Measurements

Development stages for each cultivar were recorded regularly to provide an accurate timing for destructive plant sampling. Two plant samplings were conducted for the cultivars according to the individual development stage (Table 1). In the first year, three of the line cultivars were not destructively sampled. Sampling was conducted at anthesis (Ant., BBCH growth stage 65, which is similar to the Zadok's scale; Hack et al., 1992) and physiological maturity (Mat., BBCH 92), by randomly cutting a fixed number of fully developed fertile shoots out of each plot at ground level. In the second year, N1 was not destructively sampled. At anthesis, 30, 20, and 30 and at maturity 30, 30, and 60 fertile shoots per plot were selected in the first, second, and third year, respectively. Boundary rows were excluded for plant sampling. No differentiation between main shoots and primary tillers was made. The plant material was manually separated into leaves, stems including leaf sheaths, and spikes. In 2015, additional sampling was conducted at milk and dough ripeness by cutting 20 shoots per plot. At maturity, milk and dough ripeness, harvested spikes were additionally separated into grains and chaff using a stationary thresher. Plant samples of leaves, stems, and spikes (chaff) were oven dried at 50°C until a stationary weight was reached for subsequent determination of dry weight. The samples were ground to detect the N content by mass spectrometry using an Isotope Radio Mass Spectrometer with an ANCA SL 20–20 preparation unit (Europe Scientific, Crewe, UK) in the first year. In the second and third year, near-infrared spectroscopy (NIRS) using a FOSS NIRS 6500 (NIR System, Silver Spring, Md.) and an FT-NIRS (Bruker, MPA, Billerica, Mass.) was used instead. For NIRS analysis, vegetative plant samples were homogeneously ground using a 1 mm sieve, and grains were analyzed as complete kernels. After plant sampling at maturity, all plots were mechanically harvested using a combine harvester and the grain yield of each plot was determined. Subsamples were taken from harvested grains and oven dried at 50°C for 5 days to determine grain dry matter content. Additionally, the thousand kernel weight (TKW) was determined for each plot. The grain number of the manually harvested shoots was counted to estimate the number of grains per spike. By incorporating the information of yield per spike and plot yield, the number of spikes per m^2^ was calculated. Nitrogen uptake (Nup) was calculated by multiplying nitrogen content (N%) by DM.

### Calculation of Derived Plant Traits

Dry matter (DM) units of all plant components corresponding to the number of sampled shoots were scaled up to kg ha^−1^ using the spikes m^−2^ values for each plot. To allow comparisons between cultivars in regard to translocation processes of assimilates and nitrogen, the following parameters were assessed:

The absolute amount of translocated pre-anthesis accumulated assimilates from vegetative plant organs into grains between anthesis and maturity in kg ha^−1^: Dry matter translocation (DMT) (Papakosta and Gagianas, 1991):

$$\begin{array}{ll} DMT={DM\left(spikes+stems+leaves\right)}_{anthesis}\\ - & {DM\left(chaff+stems+leaves\right)}_{maturity} \end{array}$$

The relative amount of translocated pre-anthesis accumulated assimilates into grains (Papakosta and Gagianas, 1991): DMT-efficiency (DMTEff):

$$\begin{array}{l} DMTEff=DMT/DM_{anthesis} \end{array}$$

Post-anthesis assimilation (PAA):

$$\begin{array}{l} PAA=DM_{maturity}-DM_{anthesis} \end{array}$$

The contribution of pre-anthesis assimilation to grain filling (CPreAA):

$$\begin{array}{l} CPreAA=DMT/{DM\left(grain\right)}_{maturity} \end{array}$$

The ratio of grain DM to total DM at maturity (Harvest index, HI):

$$\begin{array}{l} HI={DM_{grain}/DM}_{total\;} \end{array}$$

The absolute amount of translocated pre-anthesis accumulated nitrogen from vegetative plant organs into grains between anthesis and maturity in kg/ha^−1^ (NT) (Cox et al., 1985):

$$\begin{array}{ll} NT={Nup\left(spikes+stems+leaves\right)}_{anthesis}\\ - & {Nup\left(chaff+stems+leaves\right)}_{maturity} \end{array}$$

Accordingly, partial NT was calculated for spikes, stems and leaves.

The relative amount of translocated pre-anthesis accumulated nitrogen into grains (Cox et al., 1985): NT-efficiency (NTEff):

$$\begin{array}{l} NTEff=NT/Nup_{anthesis} \end{array}$$

Accordingly, partial NTEff was calculated for spikes, stems and leaves.

Post-anthesis nitrogen uptake (PANup):

$$\begin{array}{l} PANup=Nup_{maturity}-Nup_{anthesis} \end{array}$$

The contribution of pre-anthesis nitrogen to total nitrogen uptake (CPreNup):

$$\begin{array}{l} CPreNUP={Nup}_{anthesis}/{Nup}_{maturity} \end{array}$$

The ratio of grain nitrogen uptake (Nup) to total Nup at maturity (N Harvest index, NHI):

$$\begin{array}{l} NHI={Nup_{grain}/Nup}_{total\;} \end{array}$$

Apparent nitrogen uptake efficiency, calculated for anthesis and maturity as the ratio of total Nup to total N fertilized (Moll et al., 1982):

$$\begin{array}{l} NupEff={Nup_{total}/N}_{fertilized\;} \end{array}$$

The efficiency of the internal conversion of N into total DM (Nitrogen utilization efficiency, NutEff_total_) and grain DM (NutEff_grain_) (Moll et al., 1982; Lammerts van Bueren and Struik, 2017), where NutEff_total_ was calculated for anthesis and maturity.

$$\begin{array}{l} NutEff_{grain}={DM_{grain}/Nup}_{total\;}\\ NutEff_{total}={DM_{total}/Nup}_{total\;} \end{array}$$

The efficiency of the conversion of fertilized N into total DM (Nitrogen use efficiency, NUE_total_) and grain DM (NUE_grain_) (Moll et al., 1982), where NUE_total_ was calculated for anthesis and maturity:

$$\begin{array}{l} NUE_{grain}={DM_{grain}/N}_{fertilzed}\\ NUE_{total}={DM_{total}/N}_{fertilized\;} \end{array}$$

The soil N component was neglected for the calculation of NupEff and NUE.

### Statistical Analysis

Due to the lack of some cultivars in 2014 and of N1 in 2015, the plant traits were analyzed within the 3 years, considering N-level and cultivar group as main effects, their interaction and a random block effect, using the *lmerTest::lmer*-function in R. Analyses were conducted in *R* (version 3.4.2; R Core Team, 2017). Both cultivar groups were compared within N-levels by Tukey's HSD *post-hoc* test. Estimated marginal mean (emmean) trait values were calculated within N-levels in the 3 years for both cultivar groups, using the *emmeans::emmeans* function in *R*, and used for plotting the trait values. Commercial heterosis (CH) was calculated as the difference between the emmean values of the hybrid group and the line group divided by the emmean values of the line group within the 8 year^*^N-level combinations, for comparing both cultivar groups per se. CH values were ranked by traits and N-levels.

## Results

### Treatment Effects on Plant Traits

Including cultivar group and N-level as fixed effects, N-level main effects were highly significant (*p* < 0.001) for most traits (Table 2). Cultivar group^*^N-level interactions were rarely found and never for all years. In contrast, N-level effects were not significant for the DM translocation efficiency, the contribution of pre-anthesis assimilation to grain filling and the contribution of pre-anthesis N uptake to total nitrogen uptake, and for N translocation efficiency traits and N harvest index only in 2016.

**Table 2:** Evaluated traits of dry matter formation, N uptake, N content and indirect, derived dry matter, and N-traits with abbreviations.

		**ANOVA (*****p*** **<** **0.05)**									***post-hoc*** **(*****p*** **<** **0.05)**							
		**2014**			**2015**			**2016**			**2014**			**2015**		**2016**		
**Trait name**	**Abbreviation**	**N-level**	**cultivar group**	**Interaction**	**N-level**	**cultivar group**	**Interaction**	**N-level**	**cultivar group**	**Interaction**	**N1**	**N2**	**N3**	**N2**	**N3**	**N1**	**N2**	**N3**
**DRY MATTER TRAITS**																		
Spikes DM at anthesis	DM Ant. spikes	^***^	^***^				^**^	^***^	^**^				l					l
Stems DM at anthesis	DM Ant. stems	^***^	^**^					^*^					l					
Leaves DM at anthesis	DM Ant. leaves	^***^	^***^		^*^			^***^	^***^				l					l
Grain DM at maturity	DM Mat. grain	^***^			^*^			^**^	^***^									h
Chaff DM at maturity	DM Mat. chaff	^***^	^**^					^***^	^*^				l					
Stems DM at maturity	DM Mat. stems	^***^			^*^			^***^										
Leaves DM at maturity	DM Mat. leaves	^***^	^***^		^*^	^***^		^***^	^**^			l	l		l		l	l
Total DM at anthesis	DM Ant. total	^***^	^***^				^*^	^**^	^*^				l					l
Total DM at maturity	DM Mat. total	^***^			^*^			^***^	^*^									
**DERIVED DRY MATTER TRAITS**																		
Harvest index	HI		^***^			^**^		^*^	^***^		h	h			h			h
Post-anthesis assimilation	PAA	^***^	^***^		^*^				^***^		h	h	h			h		h
DM translocation	DMT	^**^	^***^						^**^				l					l
DM translocation efficiency	DMTEff		^**^						^*^		l							
Contribution of pre-anthesis assimilation to grain filling	CPreAA		^***^						^**^		l		l					l
Grain number per spike	GNS		^***^			^***^			^*^		h	h	h	h	h			
Thousand kernel weight	TKW		^***^			^*^		^*^			l		l					
spike density	spike density	^***^	^***^		^*^			^***^				l	l					
Total nitrogen use efficiency at anthesis	NUE Ant. total	^***^	^***^		^**^		^*^	^***^	^*^				l					
Total nitrogen use efficiency at maturity	NUE Mat. total	^***^			^**^			^***^	^*^							h		
Grain nitrogen use efficiency at maturity	NUE Mat. grain	^***^	^*^	^*^	^**^	^*^		^***^	^***^		h			h		h		
Total nitrogen utilization efficiency at anthesis	NutEff total Ant.	^***^			^***^	^**^		^***^	^*^					h				
Total nitrogen utilization efficiency	NutEff total Mat.	^***^	^***^		^***^			^***^	^***^		h	h	h			h	h	
Grain nitrogen utilization efficiency	NutEff grain	^***^	^***^		^**^	^*^		^***^	^***^		h	h	h			h	h	h
**N CONTENT TRAITS**																		
Spikes nitrogen content at anthesis	NC Ant. spikes	^**^			^*^	^***^		^*^	^**^					l	l			
Stems nitrogen content at anthesis	NC Ant. stems	^***^			^***^	^***^		^***^	^***^					l	l	h	h	
Leaves nitrogen content at anthesis	NC Ant. leaves	^***^	^***^		^**^			^***^				h	h					
Grain nitrogen content at maturity	NC Mat. grain	^***^	^***^		^**^	^***^		^***^	^***^		l	l	l	l	l	l	l	l
Chaff nitrogen content at maturity	NC Mat. chaff		^*^			^**^		^***^	^*^					l			l	
Stems nitrogen content at maturity	NC Mat. stems	^**^			^*^			^***^										
Leaves nitrogen content at maturity	NC Mat. leaves	^***^			^*^			^***^	^**^									h
**N UPTAKE TRAITS**																		
Spikes nitrogen uptake at anthesis	Nup Ant. spikes	^***^	^***^		^*^		^*^	^***^				l	l					
Stems nitrogen uptake at anthesis	Nup Ant. stems	^***^	^*^		^**^			^***^					l					
Leaves nitrogen uptake at anthesis	Nup Ant. leaves	^***^	^*^		^**^			^***^	^**^				l					
Grain nitrogen uptake at maturity	Nup Mat. grain	^***^	^***^	^**^	^**^			^***^				l	l					
Chaff nitrogen uptake at maturity	Nup Mat. chaff	^***^	^***^					^***^	^**^			l	l				l	
Stems nitrogen uptake at maturity	Nup Mat. stems	^***^			^**^			^***^										
Leaves nitrogen uptake at maturity	Nup Mat. leaves	^***^	^***^	^*^	^*^	^**^		^***^				l	l		l			
Total nitrogen uptake at anthesis	Nup Ant. total	^***^	^***^		^*^			^***^					l					
Total nitrogen uptake at maturity	Nup Mat. total	^***^	^***^	^**^	^**^			^***^				l	l					
Straw nitrogen uptake at maturity	Nup Mat. straw	^***^	^***^		^*^			^***^				l	l					
**DERIVED N TRAITS**																		
Post anthesis nitrogen uptake	PANup				^*^													
Contribution of pre-anthesis N uptake to total nitrogen uptake	CPreNup					^*^												
total nitrogen translocation	NT	^***^	^**^		^**^		^*^	^***^					l					
Nitrogen translocation efficiency	NTEff							^**^										
Leaves nitrogen translocation	NT leaves	^***^			^**^			^***^	^**^									l
Spikes nitrogen translocation	NT spikes	^***^	^***^		^*^		^*^	^**^					l					
Stems nitrogen translocation	NT stems	^***^	^*^		^**^			^**^					h					
Nitrogen translocation efficiency leaves	NTEff leaves		^**^			^***^			^**^			h		h		l		
Nitrogen translocation efficiency spikes	NTEff spikes							^*^	^*^								h	
Nitrogen translocation efficiency stems	NTEff stems							^***^										
Nitrogen harvest index	NHI					^*^		^**^										
Nitrogen uptake efficiency at anthesis	NupEff Ant.	^**^	^**^					^***^										
nitrogen uptake efficiency at maturity	NupEff Mat.	^***^	^***^					^***^				l	l					

*Type III-ANOVA treatment effects for estimating the effect of N-level, cultivar group and the interaction within years*:

* *(p < 0.05)*,

** *(p < 0.01)*,

*** *(p < 0.001)*.

*Colored shades and letters indicate significantly different trait values (orange l: lines > hybrids, green h: hybrids > lines) as identified from pairwise comparison of the estimated marginal means by Tukey's HSD post-hoc test within the N-levels by years*.

Both cultivar groups differed significantly within all 8 year^*^N-level test cases only for some traits and in addition not consistently in the same direction. Significant cultivar group differences were found most often in 2014 followed by 2016 and 2015. Cultivar group effects were found for many direct and derived DM traits in 2014 and 2016, for Nup traits in 2014 and for some NC traits like grain N content in multiple years, but not for many derived N traits.

### Grain Yield and Dry Matter Allocation

**Figure 2:**
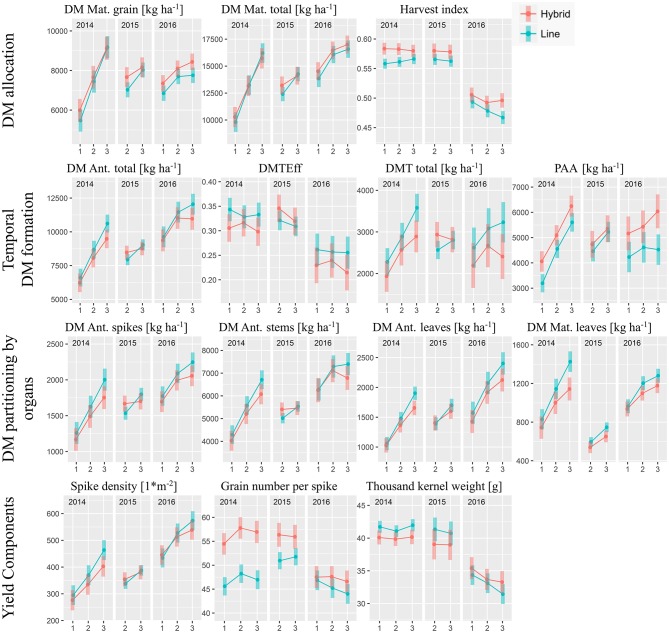
Grain yield and DM traits by cultivar groups, N-levels and years: Estimated marginal mean values with 95% confidence intervals. N-level 1, 2, and 3 refer to 100, 160, and 220 kg ha^−1^, respectively, “Ant.” to anthesis and “Mat.” to maturity.

For grain yield (DM Mat. grain), the differences in the hybrid group compared to the line cultivars, referred to as “commercial heterosis”, diminished with increasing N fertilization level in 2014 and 2015 but increased slightly for N3 in 2016 (Figure 2). However, the cultivar group^*^N-level-interaction was not significant in all years (Table 2). Overall, on average from the eight test cases (year^*^N-level), hybrids yielded 5.5% more grain than line cultivars (**Figure 5**). Total dry matter (DM) at maturity was not consistently higher for hybrids. Thus, the yield advantage of the hybrids was due to a mostly significantly better DM partitioning to the grain as expressed in the harvest index (on average +3.5% within the test cases; **Figure 5**). Compared to the first two years, when the harvest index ranged between 0.55 and 0.60, it substantially dropped in 2016, where a further decrease with higher N-levels was observed, especially for the line cultivars. The hybrids' higher harvest index at similar total DM was reflected especially in lower leaf DM at maturity while both groups had similar chaff and stem DM (Table 2).

### Temporal Formation of Dry Matter Traits

The overall similar DM formation was preceded by slightly lower DM formation until anthesis of the hybrids compared to the lines (−4.4%, Figure **5**), especially for the higher N-levels. In particular, hybrids reached lower leaf DM than lines (overall −7.1%). In addition, the hybrids' DM translocation efficiency tended to be lower in most cases (overall −6.1%) in 2014 and 2016, but individual cultivars within both groups varied substantially. Consequently, the DM translocation of the hybrid group was lower by on average 10.8%. In contrast, lower DM translocation was over-compensated for in all cases by increased post-anthesis assimilation (PAA, +16.5% on average).

**Figure 3:**
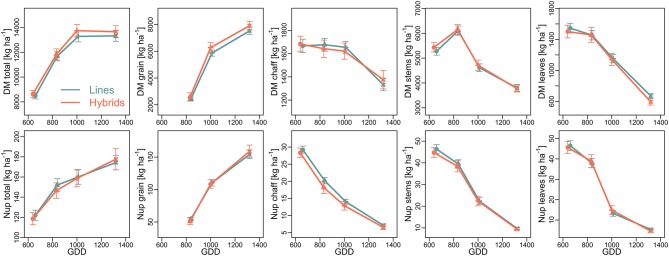
Temporal development of dry matter (DM; top) formation and N uptake (Nup; bottom) during the grain filling phase in 2015, displayed by growing degree days (GDD; 5°C threshold): Mean across two N-levels ± SE. Values are shown for anthesis (circles), milk ripeness (triangles), dough ripeness (crosses), and maturity (x).

In 2015, the additional plant sampling at milk and dough ripeness indicated similar DM formation over time for both cultivar groups during grain filling (Figure 3). The temporal development of the vegetative organs over time was comparable for both cultivar groups (Figure 3). Stem DM increased until milk ripeness before being depleted especially until dough ripeness. Chaff DM remained rather constant until dough ripeness but decreased during later grain filling, whereas leaf DM decreased almost linearly and exhibited the highest DM translocation as related to DM at anthesis. The hybrids tended to exceed the lines in total and grain DM at dough ripeness and maturity.

### Differences in Yield Components

Overall, hybrids reached higher grain yields in spite of mostly fewer spikes per m2 (−4.1%; Figures 2, **5**). The thousand kernel weight (TKW) of hybrids was lower in 2014 and 2015 but higher in 2016. The year 2016 stood out from the other years through the higher spike densities combined with low TKW and rather few grains per spike. Hybrids excelled through more grains per spike (+11.5%) especially in 2014, with the difference between cultivar groups diminishing over the years. This effect was compensated for by a more stable TKW, which, however, on average was similar for both groups.

### Nitrogen Uptake in Vegetative Organs

**Figure 4:**
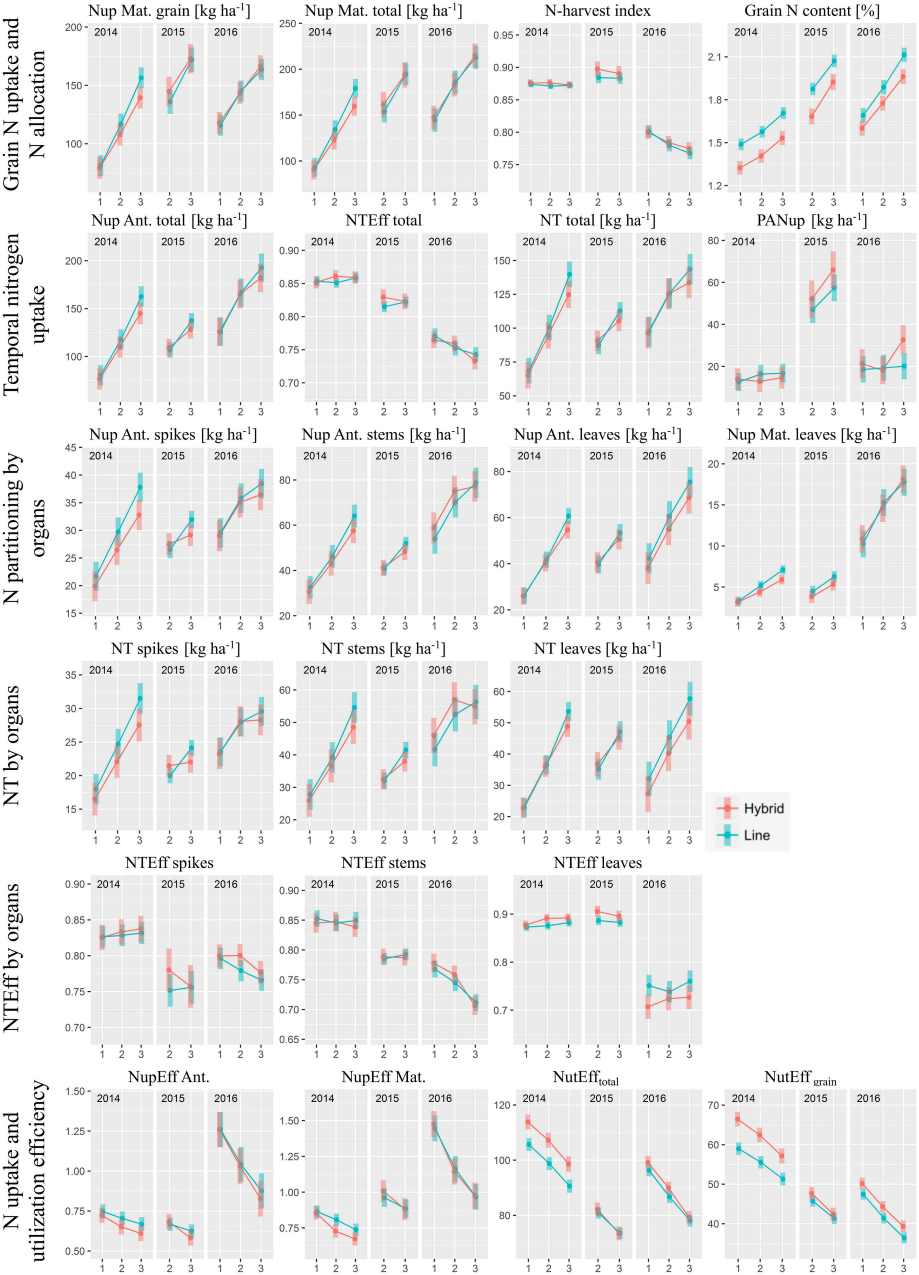
Components of nitrogen uptake (Nup) and N use efficiency by cultivar groups, N-levels and years: Estimated marginal mean values with 95% confidence intervals. N-levels 1, 2, and 3 refer to 100, 160, and 220 kg ha^−1^, respectively, “Ant.” to anthesis and “Mat.” to maturity.

**Figure 5:**
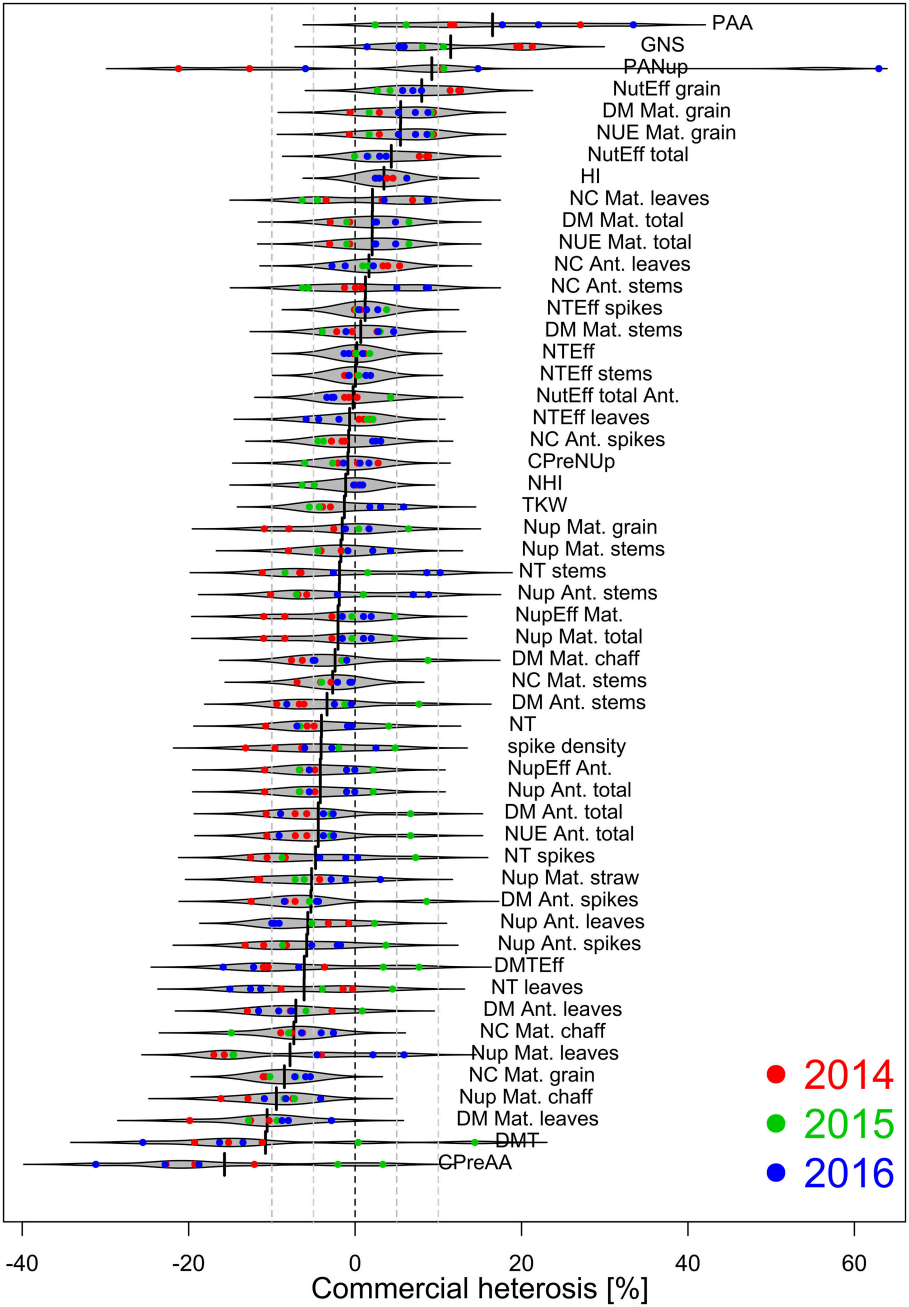
Commercial heterosis [%] by traits based on estimated marginal means within year^*^N-level combinations (*n* = 8), sorted descending. Heterosis estimates are colored by years. Negative and positive values indicate on average lower and higher trait values for hybrids than for lines, respectively. Dashed lines indicate values of 0, ± 5 and ± 10%, respectively.

Besides DM formation, assimilation relies on sufficient chlorophyll content in leaves. The lower vegetative leaf DM of hybrids went along with slightly increased nitrogen content (on average +1.7% at anthesis and +2.1% at maturity, **Figure 5**). Still, the leaf nitrogen uptake (Nup; Figure 4) was lower for hybrids compared to lines (on average −5.7% at anthesis and −7.8% at maturity). Likewise, total vegetative N uptake at anthesis (−4.2%) and total Nup at maturity (−2.1%) tended to be (mostly not significant) lower for hybrids, mainly due to differences in 2014. In contrast, year and N-level effects were dominant for the anthesis N uptake efficiency, which was on similar, rather low levels in the first two years (0.53–0.79) compared to 2016 (0.71–1.37), where the decrease with increasing N-level was much steeper. Similar curves were found for the N uptake efficiency at maturity.

### Grain Nitrogen Uptake and Nitrogen Allocation

While in 2014 hybrids reached overall lower grain Nup, the groups were comparable in the other years (Figure 4). Overall, hybrids took up slightly less grain N by 1.5% on average over the eight test cases (Figure 5) with total Nup showing qualitatively very similar N-response curves within the respective years (Figure 4).

No significant difference was found in N partitioning to the grain (N-harvest index, NHI) between the cultivar groups from the *post-hoc* test, which explains why the response of grain Nup resembled that of total Nup. Moreover, N harvest index (as the DM harvest index) was stable across N-levels in 2014 and 2015 but decreased with fertilization rate in 2016. With hybrids achieving similar grain Nup in spite of increased grain yields, grain N content was consistently (^***^) lower by on average 8.5% compared to the line cultivars. For both groups, the values were considerably lower in 2014 compared to the following years.

### Temporal Dynamics of Nitrogen Uptake

Nitrogen translocation (NT) into the grain can be represented by multiplying Nup at anthesis by N translocation efficiency (NTEff). In contrast to DM translocation efficiency, NTEff varied little between cultivar groups, decreased over the years from on average 0.85 to 0.75 and responded to N fertilization in a comparable way as the N-harvest index (Figure 4). Thus, N translocation (on average −4.0% for hybrids compared to lines) was mainly a function of the total Nup until anthesis. Slightly lower N translocation values for hybrids mainly originated from lower anthesis Nup at the highest N-level. On the organ level, while spike and stem N translocation efficiency were mostly comparable, leaf N translocation efficiency of hybrids was generally higher in 2014 and 2015 but lower in 2016.

In 2015, the dynamics of total Nup during the grain filling phase (Figure 3) were more linear compared to total DM accumulation, with still further increases in Nup after dough ripeness in spite of stagnating total DM. Starting from slightly lower anthesis Nup, hybrids tended to take up more N post anthesis, after dough ripeness. N translocation was more apparent during the early grain filling phase compared to DM translocation as visible from the steeper decrease from all vegetative organs, irrespective of cultivar groups.

### Commercial Heterosis

Relative commercial heterosis (CH) was calculated for 53 considered DM- and N-related traits within each year^*^N-level combination (Figure 5). In all years, CH, irrespective of considered traits, decreased with higher N fertilization (Supplementary Figure 1). Only in 2016, the effect was not significant due to two strong positive outliers (post-anthesis assimilation and post-anthesis Nup) in N3. However, positive median CH values across traits were only found in 2015 in N2 and in 2016 in N1.

The traits were ranked by commercial heterosis within the 8 year^*^N-level combinations (Figure 5). Most noticeable, post-anthesis assimilation (PAA), grain number per spike (GNS), and post-anthesis Nup (PANup) reached the highest CH values, but CH for PANup differed substantially between years and N-levels. CH ranged from about 0 to +10 % for grain nitrogen use efficiency (NUE Mat. grain), identical as for grain DM at maturity, total nitrogen utilization efficiency, and harvest index (HI). The lowest CH was observed for the contribution of pre-anthesis assimilation to grain filling (CPreAA) and DM translocation, with values down to −20% for both traits followed by leaf DM at maturity, chaff Nup at maturity and grain N content at maturity.

## Discussion

### Heterosis for Grain Yield Formation

Effects of the years and different nitrogen fertilization influenced the observed dry matter and nitrogen traits of hybrid and line cultivars. The average grain DM of 7.6 t ha^−1^ across years and treatments corresponds to regional yield levels. The N fertilization effect for grain DM was highest in 2014, due to the low soil N supply together with the overall most favorable growing conditions and the highest fungicide intensity among the 3 years. Moreover, the first and second N applications in 2014 were 2 to 3 weeks earlier than in 2015 and 2016. This effect is likely to have increased the N effect on grain DM (Bodson et al., 2001; Efretuei et al., 2016) but reduced the grain N content to much lower values than in the following years through a dilution effect (Stewart and Dwyer, 1990; Oury and Godin, 2007). In contrast, in spite of similar grain DM in N2 in all years, it increased less for N3 in 2015 and 2016 than in 2014, possibly due to the drought/heat effect in 2015 and the high pathogen pressure in 2016. The flat N response curve for grain DM in 2016, originating from a much higher level in N1 than in 2014, relates, to the higher soil N supply. Still, total DM values in 2016 exceeded those of 2014 by on average 2.3 and 2.5 t ha^−1^ at maturity and at anthesis, respectively, suggesting very favorable growing conditions during the vegetative phase. During grain filling, however, the dense canopy, together with frequent precipitation events led to visible leaf diseases, which were not sufficiently prevented through fungicides. Moreover, the preceding crop winter wheat caused fusariosis and stem diseases, and it is likely that pathogens were enhanced under higher N-levels (Bancal et al., 2007; Fagard et al., 2014). As a consequence of this putatively detrimental effect, grain yield almost stagnated for most line cultivars but still increased for the hybrids by on average 0.5 t ha^−1^, possibly suggesting a better tolerance of the hybrids to biotic stress.

The average commercial heterosis for grain DM of 5.5% in this study was lower than in many previous studies. Similar commercial heterosis was already reported two decades ago (Bodson et al., 1997). However, it varied strongly in other publications and was often higher when evaluated on the parental performance (Longin et al., 2013) or in stress-prone environments. Lower heterosis may be found under favorable growing conditions (Morgan et al., 1989; Bodson et al., 2001; Kindred and Gooding, 2005; Noorka et al., 2013) whereas heterosis beyond 10% was mostly reported from drought-influenced environments. However, more recent hybrid genotypes indicated higher heterosis under German conditions as well (Thorwarth et al., 2018). Our results only partly support the higher heterosis reported under low compared to high N conditions (Le Gouis et al., 2002). The slightly higher commercial heterosis (CH), both for grain and total DM and for the harvest index in the lower N-levels in 2014 and 2015, may indicate some advantage of the hybrids for DM production and partitioning in nutrient-limited environments, which, however, vanished in N3. Unlike for the N-limited cases, higher CH for grain DM in N3 in 2016 was not due to an increased CH for total DM but to a more stable harvest index.

The hybrids' higher post-anthesis assimilation is in agreement with previous findings (Oury et al., 1993), indicating similar translocation but accelerated and partly prolonged grain filling. Interestingly, the hybrids' higher capacity for post-anthesis assimilation was not associated with higher leaf DM or Nup.

Oury et al. (1993) found positive grain yield heterosis due to heterosis in total biomass at a comparable harvest index, while other studies reported positive grain yield heterosis due to a combined effect of both harvest index and total biomass, but dominated by increased biomass (Morgan et al., 1989; Le Gouis et al., 2002; Kindred and Gooding, 2005). In contrast, our results indicate a dominant effect of a higher harvest index. Increasing grain yield through increasing the harvest index may be a strategy for stress resistance (Fischer and Maurer, 1978). The low use of pre-anthesis assimilation for grain filling of the hybrids may indicate persisting sink limitation in the face of higher assimilation post anthesis (Reynolds et al., 2005). However, the difference method applied for estimating the contribution of pre- and post-assimilation and N uptake is a coarse “black box” approach, neglecting the internal fluxes between organs, including roots, respiratory losses, and leaf fall, and may, therefore, overestimate the contribution of the translocation processes (Slafer and Savin, 1994). The temporal development during the grain filling phase was only monitored in 2015, the year when post-anthesis assimilation was most similar for both groups. Consequently, no clear differences in the temporal DM formation were identified this year, which is contrary to previous findings (Oury et al., 1993).

Hybrids reached high grain yield from differently weighted yield components with on average slightly fewer spikes per m^2^ and lower TKW. Grain number per spike was strongly increased, confirming previous studies (Mahajan et al., 1999; Kindred and Gooding, 2005).

The advantage in grain DM originated only partly from a better conversion of absorbed N into total DM (N utilization efficiency) but mostly from a better partitioning into the grain (harvest index), whereas the N uptake efficiency was similar for both groups.

### Nitrogen Allocation

In contrast to the line cultivars, none of the hybrids was grouped as high baking quality and thus the constantly lower grain N content is not surprising and indicates that current hybrid cultivars are not consistently able to increase the grain protein deviation (Oury and Godin, 2007; Thorwarth et al., 2018). However, hybrids appear not to have a lower baking quality per se (Mahajan et al., 1999; Thorwarth et al., 2018).

The relative N fertilization effect for grain N content was comparable for both cultivar groups. Decreasing the N surplus is a major target in current plant breeding. Strong Nup together with strong partitioning into the harvested product (N harvest index) are desirable (Garnett et al., 2015; Cormier et al., 2016). Moreover, early Nup may be more reliable and increases the conversion efficiency into biomass. The results confirm the well-known decrease of N uptake efficiency with higher fertilization intensity (Latshaw et al., 2016; Lammerts van Bueren and Struik, 2017) but management and year effects were considerable.

Unlike grain DM, neither total nor grain Nup were higher for the hybrids, rejecting the hypothesis of higher N uptake efficiency, both for grain and total Nup. At the same time, unlike for the DM harvest index, the group differences in N harvest index were negligible. Previously, little mid-parent (Kindred and Gooding, 2004) or commercial (Bodson et al., 1997) heterosis for total Nup was reported, but grain Nup was increased through a higher N harvest index (Kindred and Gooding, 2004). However, recently, the best experimental hybrids indicated potential for increasing the grain Nup (Thorwarth et al., 2018).

The later application of the second and third dressings in 2015 strongly increased PANup in 2015. Overall, the higher post-anthesis assimilation of the hybrids was much less reflected in increased PANup than previously reported for hybrids in comparison to their parents (Oury et al., 1995). Moreover, the temporal dynamics during grain filling in 2015 did not indicate a fundamental difference between the cultivar groups. In addition, an advantage of the hybrids for total or organ-specific pre-anthesis Nup was not found, thus not supporting different splitting of the nitrogen dressings for hybrids as previously suggested (Bodson et al., 2001).

The year 2016 was characterized by considerably lower depletion of the vegetative nitrogen uptake, resulting in low values of N harvest index and N translocation efficiency, especially with increased fertilization. Visible leaf and culm diseases are likely to have affected the remobilization and transportation of nitrogen into the grain. Reversely, especially residual leaf N content at maturity (not shown) was 2-3 times higher than in 2014. Thus, adapted fungicide treatments appear important for maintaining overall Nup, N translocation and N harvest index (Ruske et al., 2003), irrespective of cultivar groups as suggested by similar NTEff values.

### Commercial Heterosis by [Test Conditions]

The results indicate positive CH for only a few plant traits. Across all traits, a tendency to higher CH under N limited conditions could be found, but this only relates to better N utilization efficiency and advantages in DM partitioning, not to better N acquisition of the hybrids. Though commonly hypothesized, heterosis was not always higher under stress conditions (Jordaan, 1996; Kindred and Gooding, 2005; Noorka et al., 2013).

## Conclusions

This study compared the groups of high-yielding commercial line and hybrid winter wheat cultivars under contrasting N fertilization in Western Europe. We found positive commercial heterosis for grain yield (+5.5 %), but the tested hybrids reached a lower grain N content (−8.5%). Thus, no advantage was found in N acquisition, not even for lower N levels and not for N partitioning. Still, hybrids showed an increased N utilization efficiency and mostly excelled through their higher grain harvest index and their high grain number per spike, which may be a greater advantage under drought conditions. The estimated heterosis does not exceed findings from older studies, indicating that the progress in line breeding during the last two decades was able to keep up with the attempts made for boosting hybrid cultivars. Given the variation and degree in yield heterosis, it currently appears to be too low to compensate for high seed costs under comparable conditions. However, the hybrids' competitive advantage will also depend on the distribution of available reference line cultivars, the agronomic management with respect to seeding density, target grain quality and its payment, or fertilization strategies. Therefore, comparing the cultivar groups in further environments including more genotypes will be necessary. Moreover, hybrid breeding benefits from increased investments in recent years and is likely to show higher effects in less favorable wheat growing regions.

## Author Contributions

SK, YH, LP, and US conceived and designed the experiments. LP and SK performed the experiments. LP analyzed the data. LP and US wrote the paper.

### Conflict of Interest Statement

The authors declare that the research was conducted in the absence of any commercial or financial relationships that could be construed as a potential conflict of interest.

## Supplementary Material

Supplementary Figure 1 Commercial heterosis [%] by year^*^N-level combinations instead of plotted by traits as in Figure 5: Groups (*p* < 0.05) were retrieved from Tukey's HSD *post-hoc* tests within the 3 years for comparing the respective N-level effects. Boxplots including outlier values (hollow circles) were complemented by the individual values (full circles, *n* = 53).
