# Supplementary figures and images for: Nitrogen Use Efficiency and Carbon Traits of High-Yielding European Hybrid vs. Line Winter Wheat Cultivars: Potentials and Limitations
Source: Front Plant Sci. 2019 Jan 17;9:1988. doi: 10.3389/fpls.2018.01988 (PMC6344469; doi:10.3389/fpls.2018.01988)

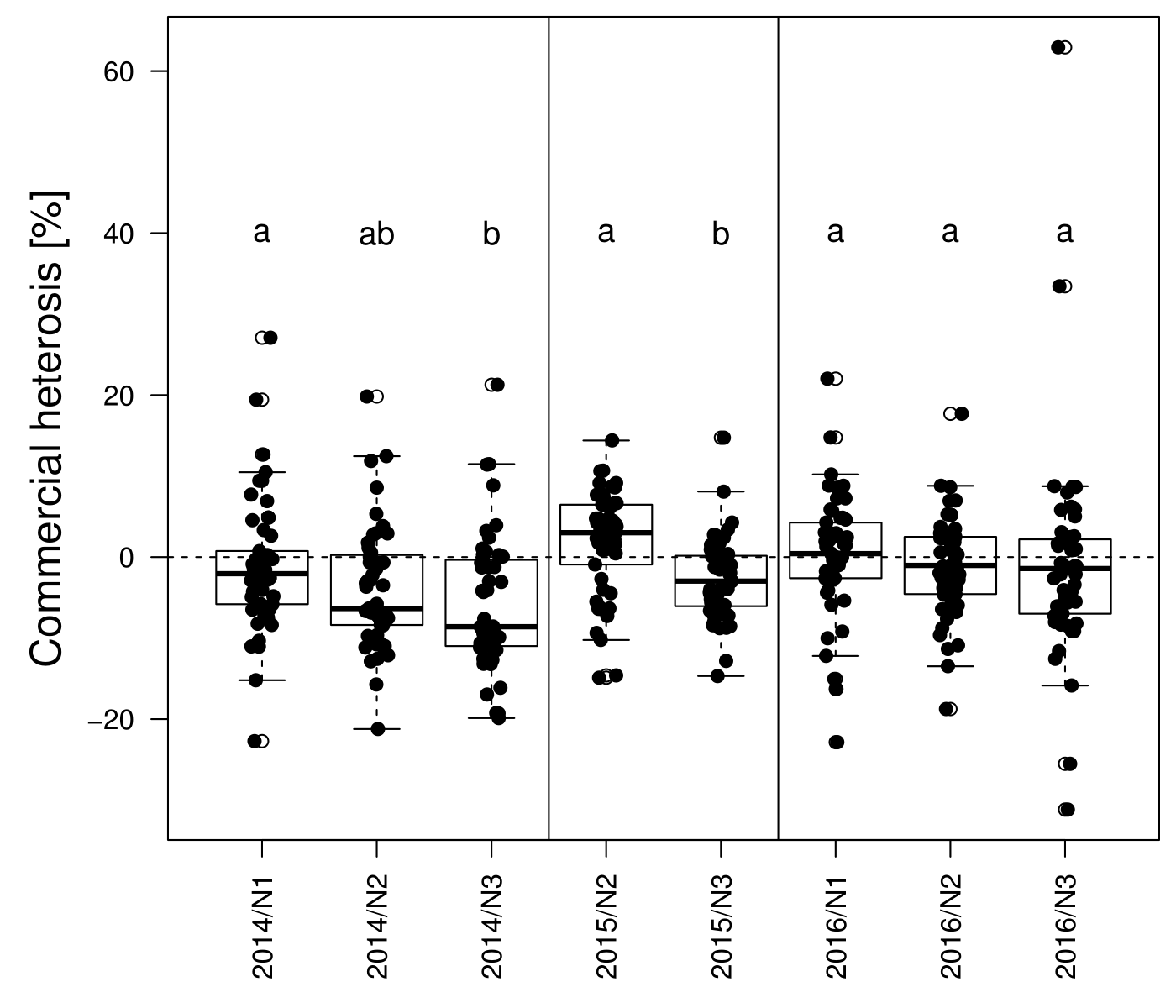

Supplement: Supplementary Figure 1 — Commercial heterosis [%] by year*N-level combinations instead of plotted by traits as in Figure 5: Groups (p < 0.05) were retrieved from Tukey's HSD post-hoc tests within the 3 years for comparing the respective N-level effects. Boxplots including outlier values (hollow circles) were complemented by the individual values (full circles, n = 53). [file Image_1.TIFF]
